# *Chlamydia trachomatis* as a Current Health Problem: Challenges and Opportunities

**DOI:** 10.3390/diagnostics12081795

**Published:** 2022-07-25

**Authors:** Rafaela Rodrigues, Carlos Sousa, Nuno Vale

**Affiliations:** 1 OncoPharma Research Group, Center for Health Technology and Services Research (CINTESIS), Rua Doutor Plácido da Costa, 4200-450 Porto, Portugal; rafaela24sofia@hotmail.com; 2 Molecular Diagnostics Laboratory, Unilabs Portugal, Centro Empresarial Lionesa Porto, Rua Lionesa, 446 C24, 4465-671 Leça do Balio, Portugal; carlos.sousa@unilabs.com; 3 CINTESIS@RISE, Faculty of Medicine, University of Porto, Alameda Professor Hernâni Monteiro, 4200-319 Porto, Portugal; 4 Department of Community Medicine, Health Information and Decision (MEDCIDS), Faculty of Medicine, University of Porto, Alameda Professor Hernâni Monteiro, 4200-319 Porto, Portugal

**Keywords:** *Chlamydia trachomatis* infection, medical screening, diagnostic, vaccine development, epidemiology, tumorigenesis, therapeutic strategies, infectious disease, infertility, clinical decision

## Abstract

Chlamydia is one of the most common sexually transmitted bacterial infections (STIs) worldwide. It is caused by *Chlamydia trachomatis* (CT), which is an obligate intracellular bacterium. In some cases, it can occur in coinfection with other parasites, increasing the pathologic potential of the infection. The treatment is based on antibiotic prescription; notwithstanding, the infection is mostly asymptomatic, which increases the risk of transmission. Therefore, some countries have implemented Chlamydia Screening Programs in order to detect undiagnosed infections. However, in Portugal, there is no CT screening plan within the National Health Service. There is no awareness in the general healthcare about the true magnitude of this issue because most of the methods used are not Nucleic Acid Amplification Technology-based and, therefore, lack sensitivity, resulting in underreporting infection cases. CT infections are also associated with possible long-term severe injuries. In detail, persistent infection triggers an inflammatory milieu and can be related to severe sequels, such as infertility. This infection could also trigger gynecologic tumors in women, evidencing the urgent need for cost-effective screening programs worldwide in order to detect and treat these individuals adequately. In this review, we have focused on the success of an implemented screening program that has been reported in the literature, the efforts made concerning the vaccine discovery, and what is known regarding CT infection. This review supports the need for further fundamental studies in this area in order to eradicate this infection and we also suggest the implementation of a Chlamydia Screening Program in Portugal.

## 1. Introduction

According to the WHO, in 2020, 129 million new infections of *Chlamydia trachomatis* (CT) were estimated. Chlamydosis is one of the most common sexually transmitted infections (STIs) worldwide. It is caused by the pathogen *Chlamydia trachomatis*, which can be found in the mouth, penis, vagina, or anus [1]. This gram-negative bacterium is associated with 19 serovars (A, B/Ba, C, D/Da, E, F, G/Ga, H, I/Ia, J, K, L1, L2, L2a, and L3) and variants that are classified according to *ompA* genotyping. In detail, serovars A–C are associated with trachoma; serovars D–K are associated with oculogenital infections; and serovars L1–L3 are related to lymphogranuloma venereum [2]. This diversity, concomitantly with the genetic variability of the infected individuals, may lead to different clinical symptoms of infection [3,4,5,6]. Notwithstanding, in the vast majority of cases (more than 80%), the infection is asymptomatic, meaning that the individuals are unaware that they can infect others, which increases the risk of further infections [1,7,8]. Despite it being curable with the use of antibiotics, when not treated, Chlamydia can lead to long-term severe injuries to the reproductive organs, such as chronic pain, pelvic inflammatory disease, increased risk of ectopic pregnancies, neonatal injuries by vertical transmission (conjunctivitis and/or pneumonia), and can also cause infertility in women, and can cause urethritis, epididymitis, prostatitis, proctitis, and reactive arthritis in men [1,4,9,10,11]. Importantly, a possible coinfection with other microorganisms, such as human papillomavirus (HPV) [12,13], *Mycoplasma genitalium* [14,15], and *Neisseria gonorrhoeae* [16,17] has been reported. An association between Chlamydia and cervical intraepithelial neoplasia has also been reported [18]. Indeed, the worst consequences concern women’s health [19]. This evidence highlights the need to develop strategies to combat this public health problem, which is preventable. However, despite the efforts of the scientific community to develop a vaccine against this pathogen, no real solutions of this type are currently available. Therefore, in the absence of a vaccination plan, one of the most effective methods to fight against CT infection is the screening of asymptomatic women and men and achieving an accurate diagnosis in order to treat the infection effectively with the appropriate drugs, avoiding antibiotic resistance and reinfection by the partner [20,21]. Altogether, these approaches will break the transmission chain and prevent serious sequels. Regarding the detection methods, Chlamydia is not effectively detected by the conventional methods (using culture) because, despite the associated higher specificity, its sensitivity has a range from 3.9 to 80%, which is related to false negatives (~39%) [22]. Moreover, using cultures is expensive, requires approximately three to seven days, and is not technically simple. In contrast, other methods better fit the associated financial, time, and technical requirements [23]. Nucleic Acid Amplification Technologies (NAATs), such as PCR, are a good example of this. NAATs have more sensibility (~98.8%) and specificity (~99.9%) associated [24] with them and may use technologies, such as the Cobas Amplicor CT/GC (Roche Diagnostics), with the following genotyping of the microorganism if it is required [24,25,26,27]. The gold-standard method for bacterium genotyping is DNA sequencing of the *ompA* gene, encoding the major outer membrane protein (MOMP) [28].

Screening is important in preventive medicine. Thus, some authors argue that the cost–benefit and the effectiveness of implementing a Chlamydia screening program covering the general sexually active population should be thoroughly analyzed [29]. Indeed, it is well documented that, when it is not treated, the costs involving Chlamydia infection and its outcomes are an economic burden. For example, in the United States, such costs were estimated at USD 2 billion before the implementation of the Chlamydia Control Program [24,30]. The data from 2016/2017 indicate that the cost of Chlamydia treatment was USD 151 per patient [31]. It should be noted that in 1990 the estimated costs for pelvic inflammatory disease caused by CT, and its complications (such as infertility), cost USD 4.2 billion [32]. Even though this number has decreased over the years with the implementation of Chlamydia screening programs, the expenditure is still striking [33].

In this review, we investigate the available data regarding *Chlamydia trachomatis* impacts, the accuracy of the current methods that are used for its detection, the immune response to this infection, and how it affects vaccine development. Finally, we further investigate the possible severe outcomes of Chlamydia and suggest the implementation of the Portuguese Screening Program for Chlamydial infections.

## 2. *Chlamydia trachomatis* Epidemiology and Screening Programs

Chlamydia is linked to high social and financial medical costs. Thus, over the 21st century, several governments (England, Australia, Netherlands, and Sweden) have implemented a National Chlamydia Screening Program, which was designed with different strategies that have undergone alterations in order to reach the main objectives of reducing the infection transmission and the overall prevalence in the population [34,35].

The design of a screening program must be well studied in order to achieve the best results in decreasing the prevalence. Accordingly, such a program must cover sexually active individuals at ages at which the possible clinical interventions regarding the infection sequels can reach the positive effects of this approach [36]. The evidence supports the thesis that the screening age may be under 25 years old [37]. Currently, England’s National Chlamydia Screening Programme is subject to alterations concerning its aims [35]. Moreover, the studies comparing self-collection specimens (postal screening) vs traditional collection in a medical environment show that self-collection increases the screening rates; as individuals are more susceptible to this approach because it is less invasive and more convenient [38,39]. These specimens could be urine or vaginal swabs, but not blood, as stated by Hoenderboom and colleagues [40].

Despite the controversy concerning the cost–benefit and effectiveness of these screening programs, some authors argue that the implementation is favorable to the cost-effectiveness in the UK [27,29,41]. Retrospective studies on the Chlamydia screening program that was implemented in Sweden show a decline in the number of new cases in the very first phase. However, the detection increased once the most accurate detection method, PCR, was implemented, proving that the conventional methods (culture) of Chlamydia detection should be replaced by faster, easier, and more sensitive molecular biology techniques [25]. Moreover, other studies report that the number of Chlamydia infections is higher than expected, evidencing that screening must be implemented regardless of the estimated prevalence [42,43]. In addition, several studies have reported that Chlamydia is more prevalent in adolescent and young women due to their higher biological susceptibility and behavior, supporting the implementation of a screening program for Chlamydia, at least in women who are 15–24 years old [44,45,46,47]. This evidence highlights the importance of developing a screening program in all of the developed countries, covering all sexually active individuals, and re-testing—due to possible reinfections—in order to treat the positive patients in a timely manner, to control the spread of Chlamydia infection in the population, and to avoid the morbidity of those infected [4]. For a screening program to be successful, noninvasive screening methods must be used. Postal delivery tests could reach more individuals in order to better establish the population’s prevalence/incidence rate and to plan future control measures for Chlamydia infection, raising the population’s awareness about the risk factors through prevention campaigns [47,48,49]. Interestingly, Pavlin et al. 2006, designed a study in which they could access the women’s opinions regarding the screening for *Chlamydia trachomatis*. They concluded that some factors must be covered in order to satisfy the population needs, mostly in terms of literacy about the disease, that will contribute to increasing the success of this health plan [47].

Recently, Huai and colleagues have performed a meta-analysis in order to estimate the prevalence of *Chlamydia trachomatis* worldwide. They verified that this measure varies significantly in the studied regions (Africa, America, South-East Asia, Europe, Eastern Mediterranean, and Western Pacific), with South-East Asia showing the lowest prevalence of the infection, as stated also by the WHO bulletin represented in Table 1 [50]. Subsequently, the authors have suggested that, based on the previous studies regarding the cost-effectiveness of these screening programs, Latin America and Africa are the locations where the design of *Chlamydia trachomatis* screening programs with distinct guidelines is critical [50,51].

As shown in Table 1, despite the fact that the global number of CT infections in women has decreased, when looking to the 2012 and 2016 data, the prevalence remains high. Moreover, there is a current concern regarding the prevalence in the western world [52].

## *3. Chlamydia trachomatis* Development and Immune Response

**Figure 1:**
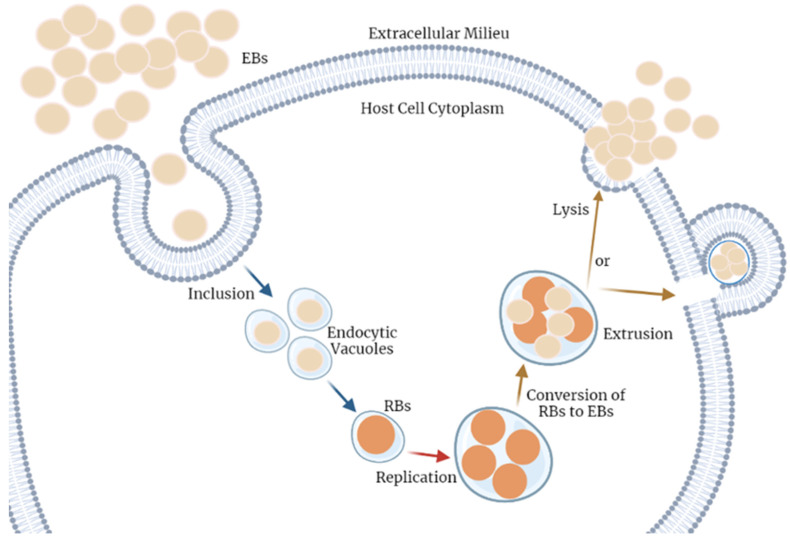
*Chlamydia trachomatis* cell cycle of infection. This pathogen alternates between two distinct forms. The infectious form, named elementary body (EB), when in contact with a host cell, can reach the cell cytoplasm by adhesion and internalization into a vacuole. Herein, EBs are converted into the alternative non-infectious form, the reticulate body (RB). These are capable of going through the replication process, using the host’s resources, and spending the cell’s energy and nutrients; concomitantly, it reaches a critical volume, thus, the RBs must transform into the previous form, EBs. Finally, there are two possible mechanisms for the extracellular EB release, (1) lysis of the host cell or (2) extrusion. This cycle occurs repeatedly in the adjacent cells. The Figure was created with BioRender.

*Chlamydia trachomatis* has a particular development biphasic cycle, as shown in Figure 1. Briefly, this pathogen alternates between two distinct forms. Firstly, the infectious form, named the elementary body (EB), which when in contact with a host cell, can be internalized into the cell cytoplasm by cell adhesion through the major out member protein (MOMP), localized into the bacterium’s envelop, and subsequent actin remodeling processes then facilitate the entry [53,54]. In order to start the reproductive cycle, EBs are converted into the metabolic active and non-infectious form, designated as the reticulate body (RB). These can go through the replication process by using the host’s resources when ATP and nutrients are available in the cell microenvironment. Otherwise, under cellular stress conditions, RBs are maintained in a reversible state of persistence. Of note, after the replication process, RBs differentiate into the previous form, EBs. For the ultimate process, the extracellular EBs are released, possibly by (1) lysis inducing apoptosis signals or (2) extrusion through exocytosis mechanisms. This cycle occurs repeatedly in the adjacent cells of the host [5,55].

Thus, this infection stimulates an immunogenic environment establishment. Notwithstanding, *Chlamydia trachomatis* has developed immune escape/evasion mechanisms. For example, it down-regulates the major histocompatibility complexes—I and II (impeding T-cell immune recognition), the modulation of specific cytokines that have pleiotropic roles (interleukin 18, beta interferon, type I interferons), and apoptosis inhibition (increasing cell survival signals and the release of Chlamydial protease-like activity factor proteins), creating a chronic inflammation with infection persistence [5,53,56,57]. In detail, this is possible due to the pathogen–host cell interactions that occur within the cytoplasm of this same cell, as well as through the modifications that this microorganism induces within its inclusion vesicles [56]. Furthermore, other immune system evasion mechanisms are well-described by Bastidas et al. 2013 [58]. Therefore, the several previously mentioned factors, mostly the adapted mechanisms of replication through the biphasic development cycle and the evolutionary protection mechanisms to surpass the milieu stress and to avoid the immune system, are the major barriers that are responsible for the challenging process of a vaccine development [54].

Nonetheless, with the improvements of the in silico studies using bioinformatic tools and machine learning predicted models, Shiragannavar and colleagues have designed a candidate vaccine that could potentially stimulate T- and B-cells for a long-term immunity establishment [59]. In addition, studies covering in silico methods, as well as immune and proteomic approaches, have been developed and have resulted in more candidate vaccines that are capable of triggering a humoral and cell response [60]. Currently, some of these studies still need in vitro and pharmacological validation, highlighting the urgent need to screen this infection in the population, aiming to eradicate it [59,61].

## 4. Chlamydia Diagnostic Methods

Chlamydia diagnosis was started by using the cell culture method. As stated previously, this method is not the most accurate one, and it is difficult to standardize. Therefore, other approaches have been developed in order to implement the most satisfactory results [62]. In line with this, antibody detection methods have been designed. For example, direct immunofluorescence (DIF), using the available kit Chlamydia Cel IF (Cellabs Pty Lty, Brookvale, Australia), and the rapid lateral immunochromatographic test (RT), using a Chlamydia test card (ulti med Products GmbH, Ahrensburg, Germany), must both be performed according to the manufacturer’s instructions. However, these tests are of the qualitative type and have an unacceptable sensitivity, meaning that they are not recommended for diagnosing this infection. In addition, serological tests that are based on the quantification of IgG, IgA, and IgM were developed using commercial ELISA (enzyme-linked immunosorbent assay) kits, namely RIDASCREEN^®^Chlamydia trachomatis, KGM2901, (R-Biopharm, Darmstadt, Germany) through serum samples, determining the ratios of the immunoglobulins of interest [63,64]. These serological approaches are associated with low specificity, and some authors also defend that seropositivity was not associated with active infection [65,66]. Currently, the most suitable *Chlamydia trachomatis* detection method is based on NAAT. For this method, urethral and cervical swabs must be collected by a medical doctor, or by the individuals themselves in the case of the women’s cervical region, using a kit, such as Cytobrush Plus Medscand^®^ Medical AB, following the manufacturer’s instructions and respecting the specimen transport times [67]. The urine samples require only sterile polypropylene containers for the collection and the further centrifugation for the pellet isolation. The next steps are the nucleic acid extraction, the evaluation through a spectrophotometer, and the amplification (Real-Time PCR) in the laboratory, for example, using an automated method with an EasyMAG kit (NucliSENS^®^ easyMAG^®^, by bioMérieux, Paris, France) or (MagNA Pure, by Roche, Risch-Rotkreuz, Switzerland) with magnetic beads for the DNA extraction [68]. Indeed, in the literature, the AMPLICOR CT/NG test for *Chlamydia trachomatis* is the most recommended. In this test, the extracted target DNA is amplified by using CT-specific complementary primers, and the hybridization of the amplified DNA is made to the oligonucleotide probes that are specific to the target for further detection of the probe-bound amplified DNA by colorimetric determination [56,68,69,70,71]. Notwithstanding, there are several other commercial alternatives available, such as Abbott RealTime m2000 CT/NG (Abbott Molecular Inc. Des Plaines, IL, USA), Aptima, (Hologic/Gen-Probe, San Diego, CA, USA), BD ProbeTec ET and Qx (Becton Dickinson, Sparks, Maryland), and Xpert CT/NG Assay (Cepheid, Sunnyvale, CA, USA) [72]. The *Chlamydia trachomatis*-positive DNA samples could then be sequenced in order to find the bacterium genotype. Briefly, the microorganism genotyping is based on *ompA* sequencing, which is amplified by using the primers that have been described by Jalal and colleagues [73] and following the protocol that has been detailed by Bianchi et al. [67], in which the amplicons must be purified by using, for example, NucleoSpin^®^ Extract II (Macherey-Nagel GmbH, Germany) or the Qiaquick PCR Purification Kit (Qiagen, Hilden, Germany). Finally, the automated DNA sequencing can occur on the ABI PRISM 3100 genetic analyzer (Applied Biosystem, CA, USA) [67,74]. Next, a bioinformatic analysis must be performed in order to align the sequences of these samples with the sequences within the databases (NCBI GenBank) using bioinformatic tools, such as ClustalX 2.1 multiple aligner, in order to classify the *Chlamydia trachomatis* strain [55,67,75].

Recently, there have been advances in point-of-care Chlamydia diagnostics that are based on rapid NAAT assays. Namely, the GeneXpert assay (Cepheid, Sunnyvale, CA, USA), which has been approved by the FDA for use in endocervical and vaginal swabs and urine specimens, and was demonstrated to have higher sensitivity and specificity, as described by Brook [76,77,78]. Notwithstanding, the test requires 90 min for the diagnostic result [79]. Thus, in order to shorten the result turnaround time, a promising prototype test is being created by TwistDx (prototype TwistDx RPA assay, Cambridge, UK) based on the isothermal recombinase polymerase amplification approach, which requires about 15 min for CT detection. This prototype is still in validation tests [79,80]. However, given the possibility of faster results compared to laboratory tests, this prototype, if it is approved and commercialized, may enable the so-called “test and treat strategy”, i.e., that doctors treat CT-positive patients in the same medical appointment, potentially revolutionizing CT infection control [80].

## 5. Current Therapeutic Options and Challenges

As stated previously, no vaccines are available in order to prevent *Chlamydia trachomatis* infections. The only option is the treatment of cases based on antibiotic prescription [81]. This bacterium is treated with antibiotics, such as tetracyclines, macrolides, inhibiting protein synthesis, fluoroquinolones, rifampin, and inhibiting nucleic acid synthesis [82]. Indeed, antibiotic resistance to this pathogen is extremely rare. However, in some circumstances, it can occur [82,83].

Usually, these drugs are designed to target the bacterium in RB form. Thus, in the pathogen cycle involving RB and EB forms, there is a phase of natural resistance against these drugs, requiring prolonged treatments in order to the elimination efficacy [84]. In addition, under adverse environmental conditions (stimuli such as antibiotics or other molecules) triggering persistent infection, these drugs’ effectiveness is compromised [85,86,87,88,89], and this is a mechanism of antibiotic resistance that could be developed by these microorganisms [84,89]. Furthermore, this bacterium commonly develops antibiotic resistance through host metabolic reprogramming during the antibiotic administration [55,84,90]. Importantly, antibiotic Chlamydia treatment could increase the probability of reinfection because these individuals would be unable to develop an immune response against this infection. As a consequence, immune memory is not created, which causes the need for retesting [91].

Moreover, it is important to highlight that it must be mandatory to adequately treat the partners of positive Chlamydia individuals in order to avoid reinfections and to effectively treat these patients [92].

## 6. *Chlamydia trachomatis* as a Risk Factor for Infertility

*Chlamydia trachomatis* infection can persist in men and women in an asymptomatic manner, affecting their fertility capabilities [93]. Recent studies concerning men’s infertility have concluded that this infection can cause the obstruction of sperm transport and even potentially cause alterations in the spermatogenesis process, affecting its quality and quantity [49,93,94].

**Figure 2:**
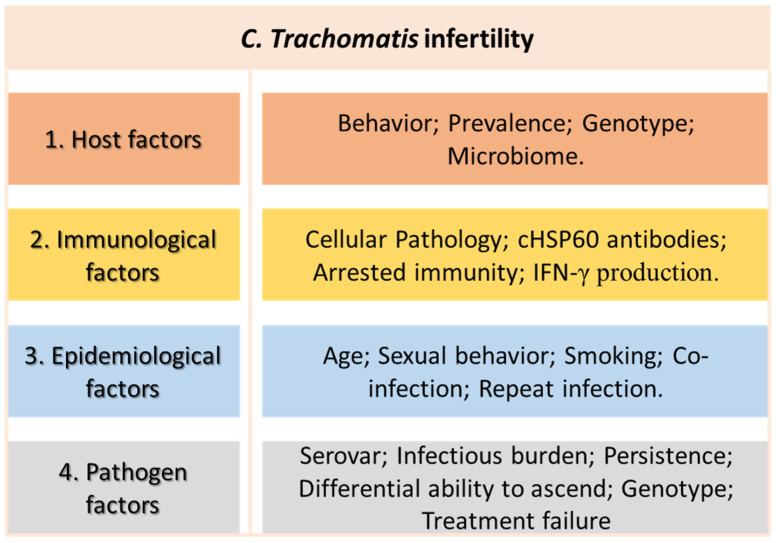
Potential factors involved in the development of Chlamydial infertility in women.

In women, this bacterium could travel from the lower into the upper reproductive tract, as described by Carey and Beagley [95], affecting the uterus, the fallopian tubes, and the ovaries, and culminating in an inflammatory disease that, depending on diverse factors, could lead to severe complications [96,97], as shown in Figure 2. Moreover, the genetic variants of candidate genes have been found to be related to the potential of the bacterium ascending within the upper genital tract and to their effect on infertility [98]. Additionally, some genetic mutations in *Chlamydia trachomatis* are reported to be associated with antibiotic (macrolides) resistance [99]. Moreover, it is well known that chronic infection by *Chlamydia trachomatis* is recognized by the upregulation of the protein Chlamydial heat-shock 60 (cHSP60), triggering the production of IgG and IgA antibodies against it, increasing the cell inflammatory response, which is essential for infection clearance. However, it may cause tissue damage, namely tubal pathology, which is a potential a risk factor for infertility, ectopic pregnancy, and other complications [7,8,100,101]. Of note, this risk is increased in cases of Chlamydia recurrence [96].

Of note, it must be highlighted that, with the ageing process, *Chlamydia trachomatis* is eliminated from the host and is no longer detected by PCR. Therefore, the effective approach to detecting past infections that may be responsible for infertility is through IgG-specific bacterium antibodies that remain over time [102].

## 7. Chlamydia and Tumorigenesis Association

To the best of our knowledge, there is no evidence regarding Chlamydia and tumors in men. On the contrary, some authors report this association in women. Paavonen and colleagues have shown that the presence of antibodies to heat shock protein-60 of *C. trachomatis* is associated with a higher risk of cervical cancer, also suggesting that the persistent infection by *C. trachomatis* is related to cervical tumors [103]. Others suggest that this protein is also involved in infertility, as previously stated by us [104].

One of the plausible molecular mechanisms that explain the association between Chlamydia infection and the increased risk of cervical cancer is based on the fact that Chlamydia infection causes an inflammatory response in the host, triggering ROS, cytokines, chemokines, growth, and angiogenic factor production, which could lead to genetic instability and abnormal mitosis [105,106]. Hanahan reports these as hallmarks of cancer and, thus, they could be responsible for cell alteration and tumor initiation [107,108,109,110]. In addition, *C. trachomatis* alters the proteins N-cadherin and beta-catenin, with important structural and regulator roles [111]. Discacciati et al. also found an important association between *C. trachomatis* and MMP-9/RECK imbalance [112]. Moreover, evidence suggests that *C. trachomatis* infection increases the risk of HPV acquisition and persistence. This coinfection increases the risk of cervical cancer because the epithelial disruption that is caused by the bacteria accelerates the virus entry, concomitantly with the weakened immune system, inducing a permissive microenvironment to cancer development [113,114,115,116,117]. Interestingly, Anttila and colleagues have found evidence concerning the highest risk for cervical cancer development and the bacterium serotype G, which is measured by the presence of IgG antibodies in the serum of Ct-infected patients [118]. Serotyping was performed through microimmunofluorescence, as described by the authors of [119,120].

As expected, because persistent inflammation is related to tumorigenesis, the ovaries, as with most organs affected by Chlamydia, can be compromised [121]. As shown by Shanmughapriya and colleagues, approximately 80% of ovarian cancer patients were diagnosed with Chlamydia [122]. In line with this, some studies have proved that anti-Hsp60 antibody concentration is increased in ovarian cancer patients [123]. Despite this evidence, there are some divergent results concerning this topic, highlighting that further studies are required in order to corroborate this association [124,125].

## 8. Discussion of the Future Perspectives concerning Chlamydia Screening Programs

We propose implementing a national health program for Chlamydia screening, using Nucleic Acid Amplification Technologies (NAATs) for higher sensitivity, as detailed by Van Der Pol and colleagues [126]. Furthermore, we suggest that further studies, such as that developed by Sabbatucci and colleagues in Italy, are still required in order to have an insight into the CT prevalence in Portugal [127].

To the best of our knowledge, only one Portuguese study from 1997, which used a limited number of samples (n = 240), has aimed to estimate the prevalence of the several genotypes of *Chlamydia trachomatis* [128]. Interestingly, in this study, the infection prevalence was 86.7%, which was probably a high-risk cohort and, therefore, not representative of the general population. With approximately the same objectives and approaches, Sylvan and colleagues [25] developed a study in Sweden that also concluded that the most frequent serovar in their cohorts was the E between both sexes, a homogeneous finding following the literature. Interestingly, in another Portuguese study by Borrego and colleagues, an uncommon prevalence of genotype H (the second most prevalent for women) was found. This genotype was not found in the Sweden study, as shown in Table 2. This might suggest that this serovar could be more frequent in Portugal than in other countries. However, no current data are available concerning Chlamydia in Portugal. Thus, more studies are required with bigger samples and different cohorts in order to reach further conclusions.

## 9. Conclusions

One of the most common sexually transmitted infections is Chlamydia, which is caused by a bacterium *Chlamydia trachomatis*, whose natural host is the human species, infecting epithelial cells in the male and female reproductive tracts. Although this infection is mostly asymptomatic, in some cases, chronic infection is associated with pelvic inflammatory disease. It can also be associated with more serious health problems, such as infertility (in both sexes) and tumorigenesis (in women). It involves social and individual problems, as well as expensive treatments and extra costs for the governments.

Therefore, it is important to control this infection and to have an insight into the real number of infected individuals and their sequels. In conclusion, this review highlights the current progress that has been made in the countries that have implemented the National Screening Program of Chlamydia regarding public health and economic concerns, avoiding the costs that are associated with the long-term injuries that are caused by this infection.

This review clarifies the impacts of Chlamydia infections on human health, suggesting that more studies should be carried out regarding the prevalence/incidence rate of the establishment of infection in Portugal in order to have an insight into this infection’s long-term injuries.

We have compared the effectiveness of the currently available and promising methods for CT detection. We have also discussed how preventive medicine measures could help to break the transmission chain in order to eradicate this infection.

Finally, our findings reinforce that, in the future, the reported evidence can drive innovation in the national health system. For example, the screening methods could be updated according to the state-of-the-art National Screening Program for Chlamydia, which could be implemented, future infection control measures could be planned, and prevention campaigns could be developed in order to raise the population’s awareness about the risk factors of this infection.

## Figures and Tables

**Table 1:** Data regarding *Chlamydia trachomatis* infection prevalence in the continents, estimates of 2012 and 2016, in women and men (Bulletin WHO) [52].

WHO Region, by Sex		
	**Chlamydia**	
	**2012**	**2016**
**Women**		
African Region	3.7 (2.7–5.2)	5.0 (3.8–6.6)
Region of the Americas	7.6 (6.7–8.7)	7.0 (5.8–8.3)
South-East Asia Region	1.8 (1.4–2.2)	1.5 (1.0–2.5)
European Region	2.2 (1.6–2.9)	3.2 (2.5–4.2)
Eastern Mediterranean Region	3.5 (2.4–5.0)	3.8 (2.6–5.4)
Western Pacific Region	6.2 (5.1–7.5)	4.3 (3.0–5.9)
Global total	4.2 (3.7–4.7)	3.8 (3.3–4.5)
**Men**		
African Region	2.5 (1.7–3.6)	4.0 (2.4–6.1)
Region of the Americas	1.8 (1.3–2.6)	3.7 (2.1–5.5)
South-East Asia Region	1.3 (0.9–1.8)	1.2 (0.6–2.1)
European Region	1.5 (0.9–2.6)	2.2 (1.5–3.0)
Eastern Mediterranean Region	2.7 (1.6–4.3)	3.0 (1.7–4.8)
Western Pacific Region	5.2 (3.4–7.2)	3.4 (2.0–5.3)
Global total	2.7 (2.0–3.6)	2.7 (1.9–3.7)

**Table 2:** Data regarding Chlamydia prevalence and the most prevalent genotypes of *Chlamydia trachomatis* (ordered by decreasing prevalence) in Portugal [128] and Sweden [25], by gender. Of note, the other genotypes (B, Ba, J, K, L1, and L2) were very rarely detected in Portugal [128].

Country	Female	Male
Portugal (n = 240)	Serovar E, H, F, G, and D	Serovar E, D/F, H, and G
Sweden (n = 449, 2 different cohorts)	Serovar E, D, F, and K	Serovar E, F, K, and D
